# Emerging MDR producing ESBL among Diarrhoegenic *Esherichia coli* from paediatric patients

**DOI:** 10.1186/1471-2334-14-S3-P70

**Published:** 2014-05-27

**Authors:** Ashita A Pinto, Yaser Siahbalaei, Arif Ali

**Affiliations:** 1 Gene Expression Laboratory, Department of Biosciences, Jamia Millia Islamia, New Delhi, India

## Background

The beta lactam antibiotics are predominant therapeutic agents against a number of infectious diseases. The production of the enzyme beta lactamase by microbes which hydrolyses the beta lactam antibiotics is an important mechanism of resistance. This study aimed to determine prevalence, phenotypic patterns, and ESBL-production status both phenotypic and genotypic for the CTX –M type ESBL in *Escherichia coli* (*E.coli*) isolated from Diarrhoeic children.

## Methods

Two hundred thirty non repetitive stool samples were collected from 2 hospitals, one private and other government hospital in Delhi, NCR. *E.coli* was confirmed by *16S* rRNA analyses. Antibiotic susceptibility and phenotypic ESBL production were studied by disc diffusion and double disk synergy tests according to Clinical Laboratory Standards Institute guidelines. PCR was performed for the molecular detection of the CTX-M type ESBL.

## Results

Out of 230, 215 were confirmed to be *E.coli*. Phenotypically 72.5% (156) showed the presence of ESBL of which 75% (79) in private hospital samples and 70% (77) in government hospital samples. PCR based molecular detection confirmed the presence of the *CTX-M* gene among 65.6% (141) of the samples.

## Conclusion

The high trends in the ESBL presence are alarming and urge means to cope with such ESBL *E.coli* strains. It being a commensal raises concern of the resistance gene transfer from pathogenic bacteria, especially in children due to common diarrhoeal episodes among them. The paediatric population is at a higher risk in case of multidrug resistance due to further limitation in therapeutic options and constrains in prescription of harsher antibiotics.

